# Supplementary material for: SARS‐CoV‐2 infection remodels the host protein thermal stability landscape
Source: Mol Syst Biol. 2021 Feb 16;17(2):e10188. doi: 10.15252/msb.202010188 (PMC7885171; doi:10.15252/msb.202010188)
Supplement: Supplementary file 1 — Appendix [file MSB-17-e10188-s001.pdf]

Appendix to
SARS-CoV-2 infection remodels the host protein thermal stability landscape

Contents

Appendix Figure S1. Comparison of centrifugation versus vacuum-based protein aggregate removal

Appendix Figure S2. SARS-CoV-2 time dependent effects on proteome thermal stability and abundance

Appendix Figure S3. GO term enrichments on 2D-TPP infection time course

Appendix Figure S4. Pathway enrichments on 2D-TPP infection time course

Appendix Figure S5. Convergence of SARS-CoV-2-induced changes to the host proteome

Appendix Figure S6. SARS-CoV-2 time dependent effects on proteome thermal stability and abundance

Appendix Figure S7. Testing of drugs and compounds that target proteins affected by stabilisation or abundance during SARS-CoV-2 infection

Appendix Figures

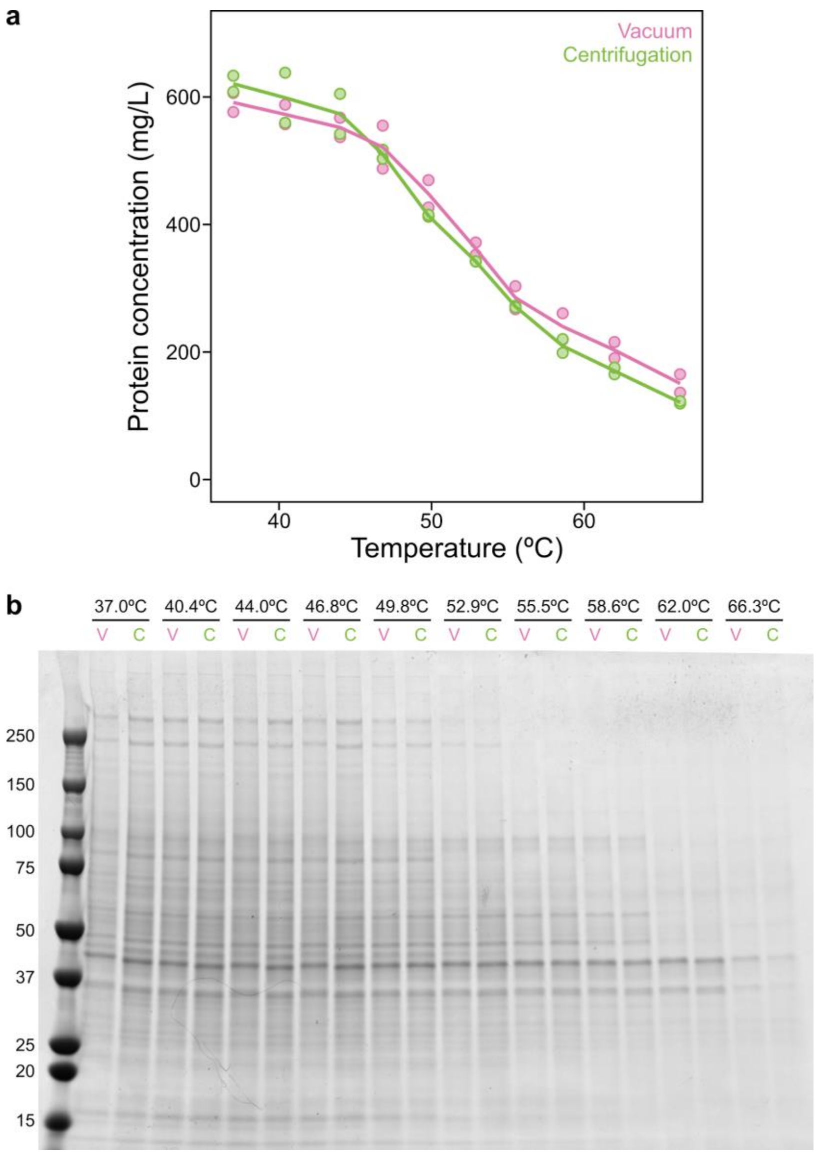

Appendix Figure S1. Comparison of centrifugation versus vacuum-based protein aggregate removal. **a**) Side by side comparison of centrifugation or vacuum manifold based removal of protein aggregates. Intact HeLa cells were subjected to 10 different temperatures and lysed with NP-40. Efficiency of protein aggregate removal through a 0.45 µM filter was compared between a vacuum manifold or centrifugation. Aggregate removal was assessed by quantifying the percentage of protein remaining soluble by BCA protein determination (n=2 technical replicates). **b**) HeLa test samples prepared according to S1A subjected to SDS-PAGE and stained with Coomassie Blue to visualize melting profiles.

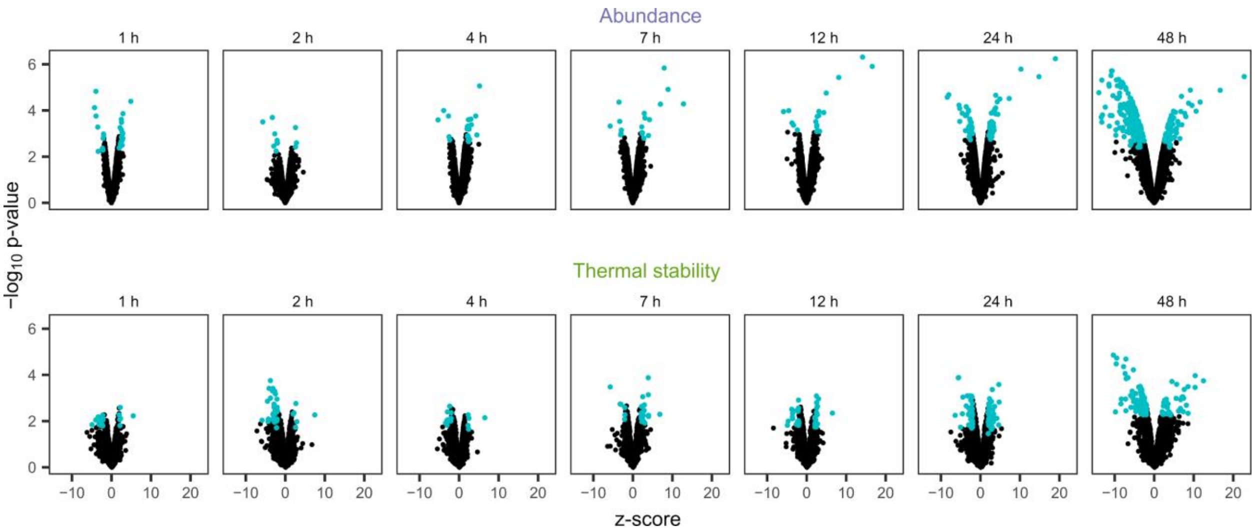

Appendix Figure S2. SARS-CoV-2 time dependent effects on proteome thermal stability and abundance. Volcano plots of all proteins detected in SARS-CoV-2 infected Caco-2 cells. Proteins classified as hits (|z-score| > 1.96 and q-value <0.05; see Methods for details) are indicated in cyan, data from three biological replicates.

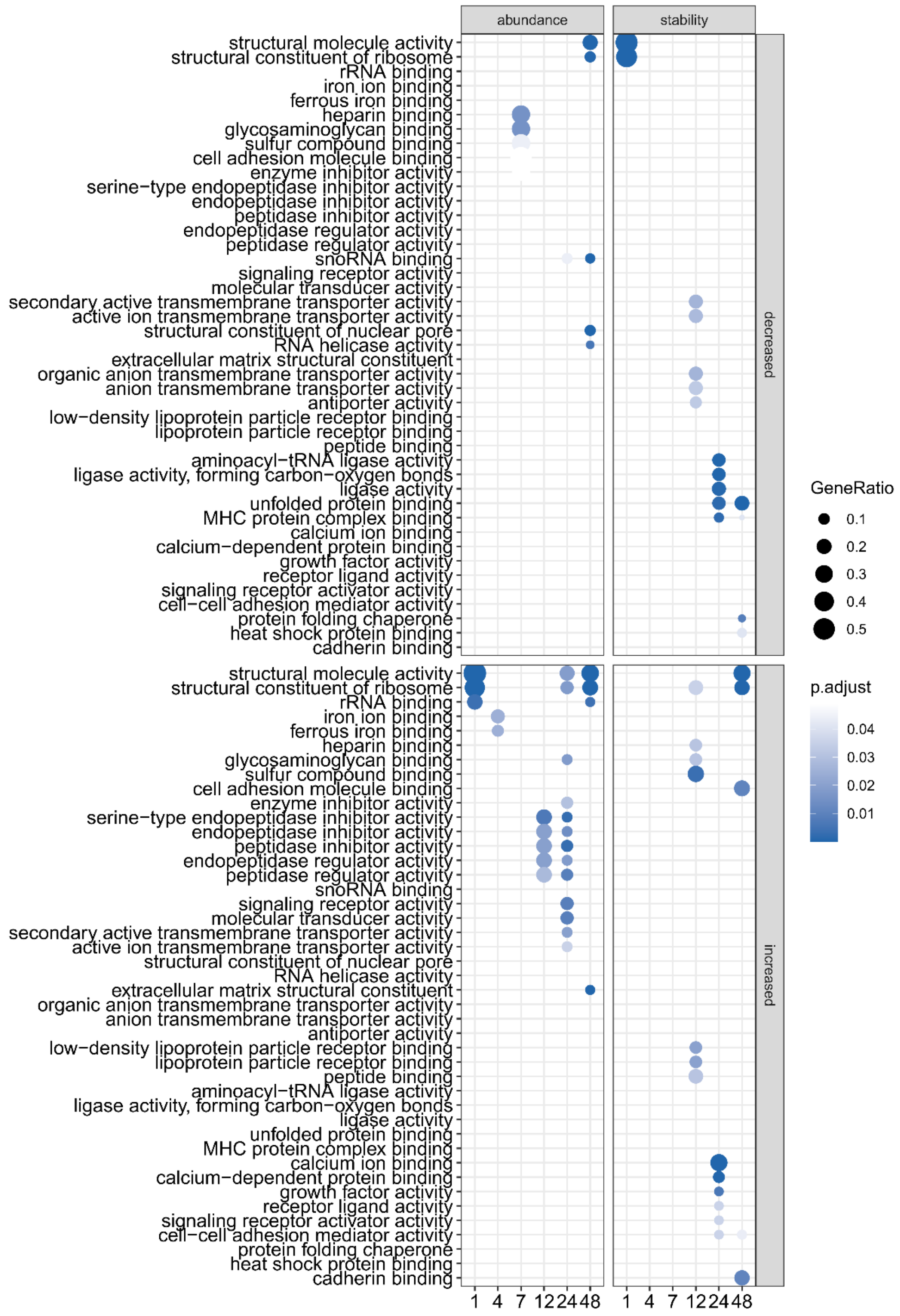

Appendix Figure S3. GO term enrichments of 2D-TPP SARS-CoV-2 infection time course. Gene Ontology (GO) enrichment of selected pathways. See Dataset EV2 for complete GO term biological process and pathway enrichments.

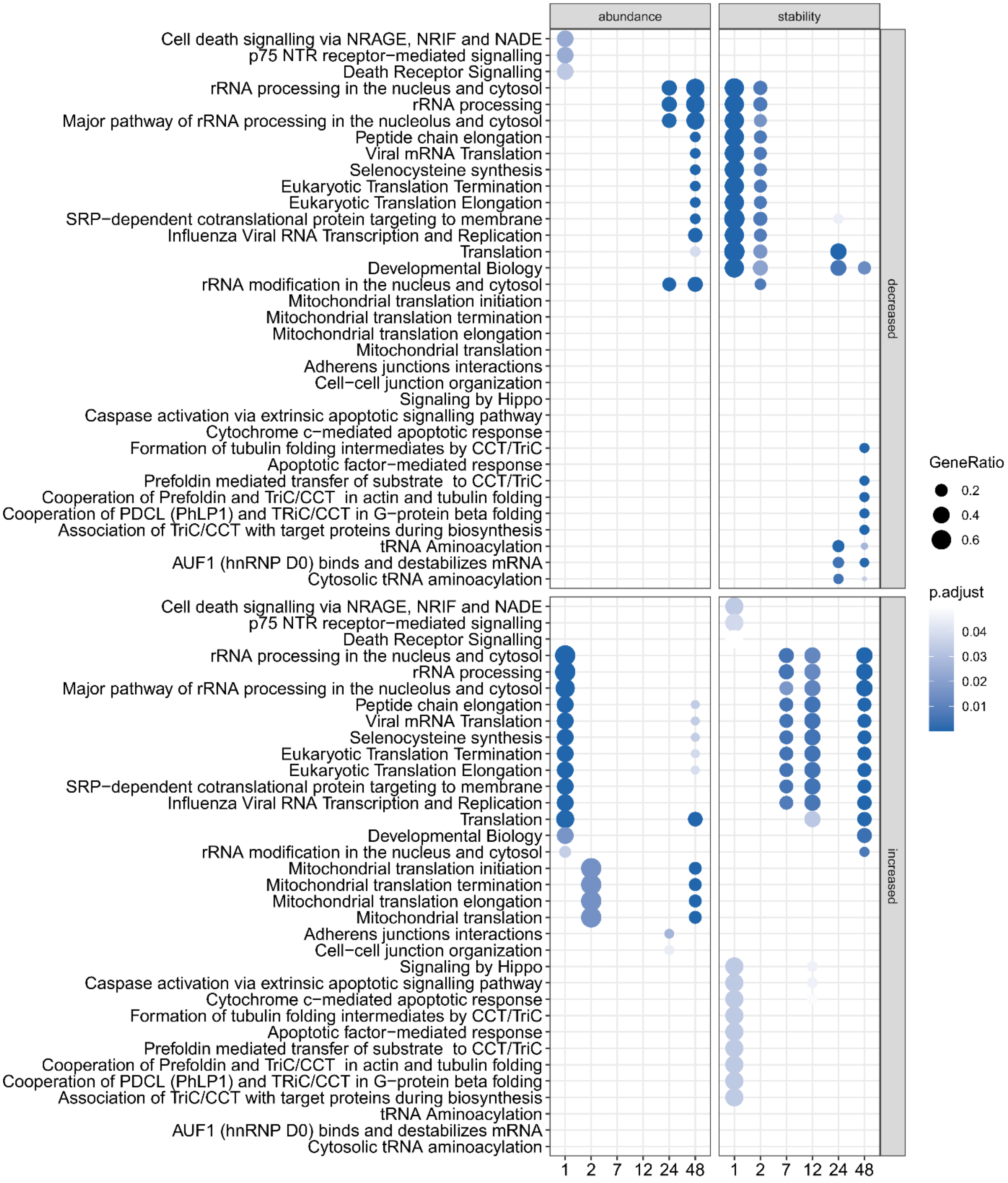

Appendix Figure S4. Pathway enrichments of 2D-TPP SARS-CoV-2 infection time course. Pathway enrichment of selected pathways. See Dataset EV2 for complete GO term biological process and pathway enrichments.

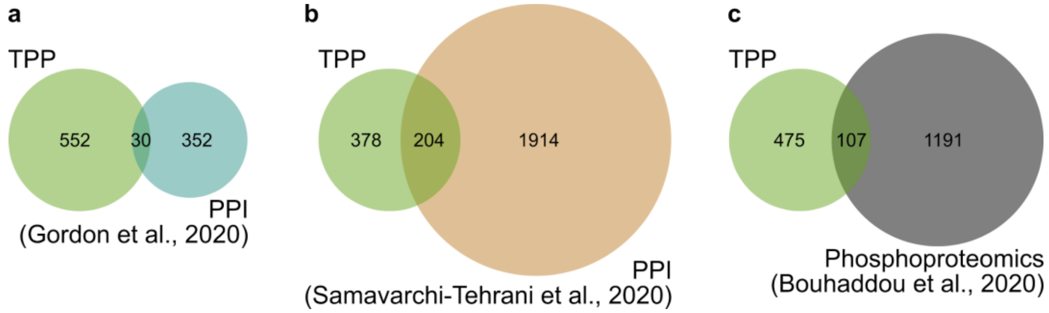

Appendix Figure S5. **Convergence of SARS-CoV-2-induced changes to the host proteome**. **a**) Venn diagram displaying overlap of proteins with altered thermostability and abundance (this work) and those that interact with SARS-CoV-2 proteins by AP-MS combined from previous work (Gordon *et al*, 2020b, 2020a). **b**) Venn diagram displaying overlap of proteins with altered thermostability and abundance (this work) and those that interact with SARS-CoV-2 proteins in BioID experiments from previous work (Samavarchi-Tehrani *et al*, 2020). **c**) Venn diagram displaying overlap of proteins with thermostability and abundance changes and previously observed changes in phosphorylation states upon SARS-CoV-2 infection (Bouhaddou *et al*, 2020).

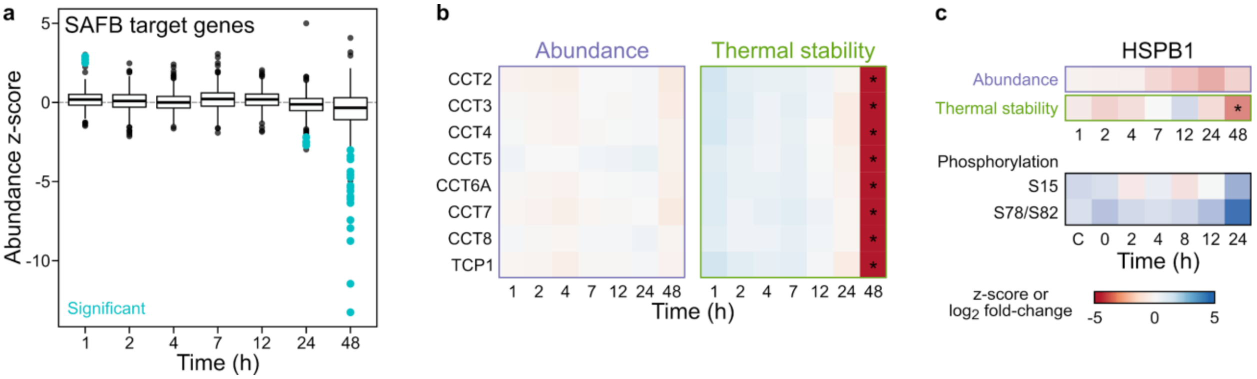

Appendix Figure S6. Thermostability changes in host proteins of significance to SARS-CoV-2. **a**) A total of 362 SAFB target genes (as defined in (Keenan *et al*, 2019)) protein abundance changes upon SARS-CoV-2 infection. Significantly regulated proteins are colored (turquoise). A total of 3 proteins were significantly upregulated at t_{1}, whereas 4 at t_{24} and 18 at t_{48} were downregulated. Centre line in box plots represents the median, box boundaries indicate the upper and lower interquartile range (IQR), and whiskers correspond to most extreme values, or to 1.5-fold of IQR if the extreme values are above this cutoff. n=3 independent biological replicates. **b**) Protein abundance and thermal stability heatmap of individual components of the chaperonin CCT complex. **c**) HSPB1 thermal destabilisation coincides with S78/82 phosphorylation events. Phosphosite data from (Bouhaddou *et al*, 2020). Note that S78/S82 could not be localized with confidence in (Bouhaddou *et al*, 2020).

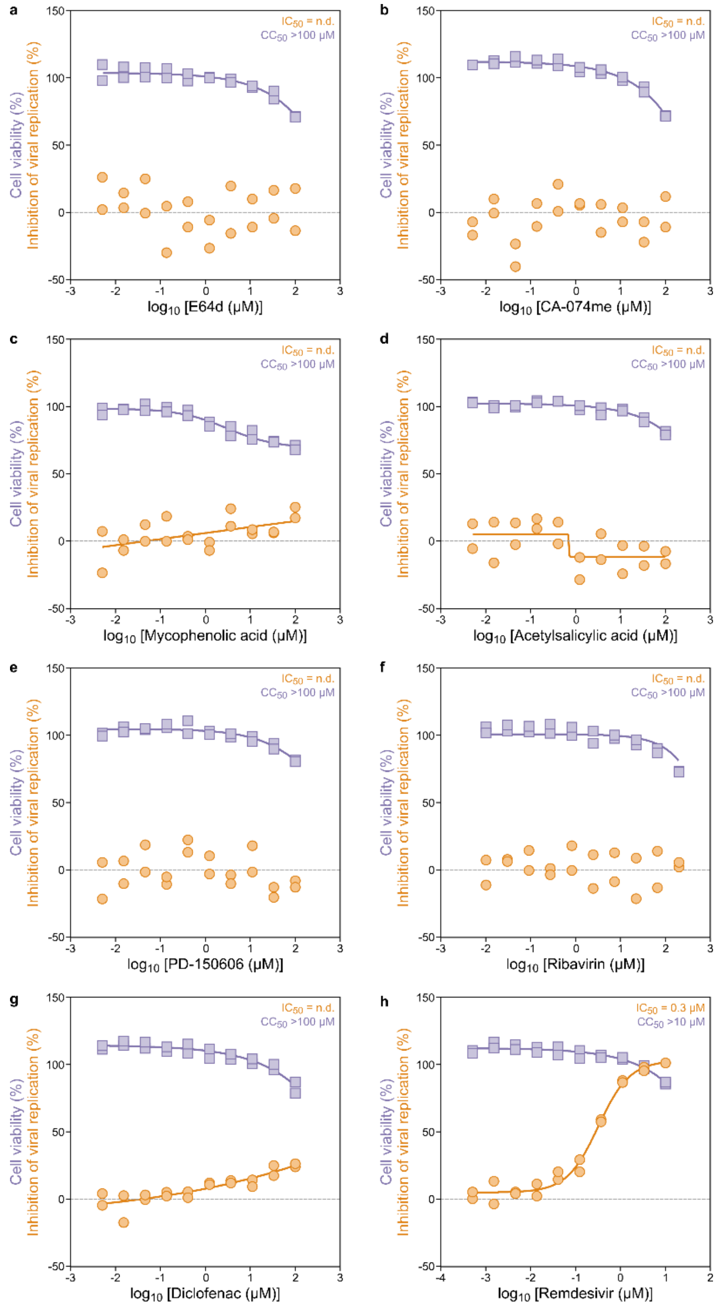

Appendix Figure S7. Testing of drugs and compounds that target proteins affected by stabilisation or abundance during SARS-CoV-2 infection. Dose response curves of compounds that inhibit activity of proteins displaying thermal stability changes in Caco-2 cells, which were ineffective or marginally affected SARS-CoV-2 infection in Calu-3 cells. **a**) E64d and **b**) CA-074me were used to inhibit cathepsins (e.g. CTSV). **c**) Mycophenolic acid used as an IMPDH Inhibitor, **d**) acetylsalicylic acid as a PTGS1 inhibitor, **e**) PD-150606 as a CAPN1 inhibitor, **f**) Ribavirin as an IMPDH inhibitor, **g**) Diclofenac as an inhibitor of PTGS1/PTGS2 (Cox-1/Cox-2) of prostaglandin metabolism and **h**) Remdesivir as an inhibitor of RNA-dependent RNA polymerase (RdRp) as an internal experimental positive control.
